# Association between calling and work engagement, and the mediating role of psychological capital and organizational commitment: a structural equation modeling study in China

**DOI:** 10.1186/s40359-026-05320-9

**Published:** 2026-08-14

**Authors:** Feifei Chang, Yunxiao Chang, Yujin Huang, Xiujie Ye

**Affiliations:** 1 Department of Internal Medicine, The First Affiliated Hospital of Guangzhou Medical University, Guangzhou, China; 2 Department of Gastrointestinal Surgery, Zhujiang Hospital, Southern Medical University, Guangzhou, China

**Keywords:** Calling, Psychological capital, Organizational commitment, Work engagement, China

## Abstract

**Purpose:**

This research aimed to examine the association between calling and work engagement, as well as the mediating roles of psychological capital and organizational commitment. The findings may inform the development of targeted interventions to enhance work engagement among clinical nurses.

**Methods:**

A cross-sectional study was conducted from November to December 2021, involving 630 registered nurses from a tertiary hospital in China. Correlation analysis and structural equation modeling were employed for data analysis.

**Results:**

Calling was positively associated with work engagement. It showed a significant direct association with work engagement, as well as indirect associations mediated by organizational commitment and psychological capital.

**Conclusion:**

Work engagement among nurses was significantly associated with calling, psychological capital, and organizational commitment. Nurse managers should set attainable goals to foster work motivation and implement appropriate strategies to enhance nurses’ sense of calling, thereby enhancing work engagement.

## Introduction

Nurses play a vital role in delivering high-quality healthcare services, which are closely associated with patient outcomes. The retention and stability of the nursing workforce are essential concerns for healthcare administrators. Maintaining low turnover and high levels of work engagement can reduce costs by improving the efficiency of recruitment, selection, and training processes [1]. In healthcare settings, low work engagement can have widespread negative effects, affecting both nurses and organizations and compromising the quality and safety of patient care [2]. In recent years, increasing attention has been paid to work engagement in nursing management, which is defined as a positive, work-related state characterized by vigor, dedication, and absorption [3]. Previous research has demonstrated that work engagement is significantly associated with positive outcomes among nurses, including lower burnout, higher organizational commitment (OC), and improved quality of care [3, 4]. Calling (CA) is defined as a sustained sense of purpose that motivates individuals to engage wholeheartedly in their work without seeking external rewards [5]. Nurses who perceive their work as a calling are more likely to view it as meaningful rather than merely a source of material gain, which is positively correlated with their dedication and linked to lower turnover intention [6].

A study involving 351 nurses found that perceiving one’s work as a CA is strongly linked to the absorption and dedication aspects of work engagement, thereby promoting active participation in daily work [7].

Additionally, individuals with a CA tend to exhibit greater organizational commitment, likely due to an enhanced sense of responsibility [8]. Higher levels of CA among staff are associated with increased OC, greater discretionary behaviors, and reduced negative emotional expressions.

As a psychological and social resource, CA provides employees with a sense of purpose and enhances their ability to cope with work-related stress and challenges [9]. Studies have shown that calling is positively associated with individual well-being, and employees with higher well-being tend to possess greater psychological capital (PC) [10].

OC is defined as an individual’s desire, obligation, or need to remain a member of an organization [11]. Furthermore, high levels of OC are considered a key characteristic of engaged nurses [12, 13]. Previous research indicates that employees with higher commitment experience less stress, demonstrate better performance, and are more likely to remain with their organization [14]. Research has demonstrated a strong association between organizational commitment and active engagement in work tasks [15].

PC is a crucial concept in positive psychology, representing a positive psychological state that individuals develop over time [16]. A growing body of research has shown that PC is significantly associated with employees’ work attitudes and performance [17, 18]. According to Luthans, employees with high PC tend to adopt a more positive outlook toward the organizational environment and work situations [19]. Previous research indicates a positive correlation between PC and work engagement, with studies showing exhibiting higher levels of work engagement when PC is elevated [20].

Although previous studies have examined the relationships among CA, PC, OC, and work engagement, significant gaps remain in the literature. First, most prior research has focused on pairwise relationships among these variables [21, 22], lacking an integrated framework to systematically elucidate the underlying mechanisms and multiple pathways through which CA influences work engagement. Secondly, in the nursing field, studies simultaneously examining the parallel mediating roles of PC and OC in the relationship between CA and work engagement are scarce [23], especially those based on rigorous theoretical frameworks such as the Job Demands-Resources (JD-R) model [24].

Therefore, this study aims to address these gaps by employing structural equation modeling to examine the association between CA and work engagement, as well as the mediating roles of PC and OC. Theoretically, this study deepens the understanding of the psychological processes and organizational attachment mechanisms through which CA relates to work engagement, and extends the application of the JD-R framework in nursing human resource management. It also provides empirical evidence for the synergistic effects of individual resources (i.e., PC) and organizational-level resources (i.e., OC) in the motivational process. The findings have important theoretical and practical implications for designing targeted intervention programs and enhancing work engagement among clinical nurses.

### Literature review

The JD-R model classifies work characteristics into job demands and job resources, encompassing two distinct psychological processes: the health-impairment process and the motivational process [25]. Job resources are positive predictors of work engagement and lead to positive outcomes by stimulating motivation, whereas the health-impairment process is associated with negative consequences such as burnout. Adequate job resources not only facilitate the motivational process and further enhance work engagement, but also contribute to favorable outcomes, including reduced turnover and increased performance [23]. As an individual internal resource, CA is manifested in perceiving work as a source of life goals and meaning [26]. Due to their strong passion and work identification, employees with a strong sense of CA are more likely to proactively engage in their work, thereby directly enhancing work engagement. Based on the above, the following hypothesis is proposed:

- H1: Calling is positively associated with work engagement.

However, the direct effect of CA on work engagement might not fully reflect the underlying mechanisms. According to the JD-R model, personal and job-related resources serve as mediators that transmit motivational effects. Accordingly, this study examines the mediating roles of PC and OC. The JD-R model elucidates a theoretical basis for understanding how PC and OC mediate the relationship between CA and work engagement. Work engagement is affected by the interplay of job demands that deplete energy, job resources that foster motivation, and personal resources such as PC and organizational commitment [24]. Job demands refer to the social, psychological, or organizational aspects of a job that require sustained effort [27]. In contrast, job resources refer to aspects of a job that help decrease the costs of job demands and promote personal growth, learning, and development [28]. The JD-R model highlights the complex interplay among job demands, personal resources, and job demands. Work engagement is shaped by both personal and job resources, which independently and interactively predict engagement levels [29].

PC among nurses is a key personal resource reflecting a positive psychological state, comprising four core components: self-efficacy, optimism, hope, and resilience, which are measurable, developable, and management [30]. These components interact synergistically, contributing to the development of both individuals and organizations [31]. PC has attracted increasing attention from researchers worldwide [32]. In recent years, numerous studies have demonstrated the beneficial effects of PC on work behaviors and occupational health outcomes among nurses, particularly in relation to work engagement. PC is not only directly associated with work engagement but also mediates the relationship between CA and work engagement among nurses [33]. This indicates that PC may serve as a valuable personal resource for enhancing work engagement [34]. Based on the above, the following hypothesis is proposed:

- H2: Psychological capital mediates the relationship between calling and work engagement.

Furthermore, OC reflects the employees’ emotional attachment to and identification with the organization [35]. Some scholars define OC as a psychological state that reflects the relationship between employees and their organization, influencing their intention to remain or leave [36]. Buchanan conceptualizes OC as a psychological bond between an individual and the organization [37]. Bateman and Strasser suggest that OC encompasses employees’ loyalty, alignment with organizational goals and values, and a desire to remain with the organization [38]. Therefore, employees with a strong sense of CA are more likely to align their professional ideals with organizational goals, thereby generating greater loyalty and enhancing OC [39].

**Fig. 1:**
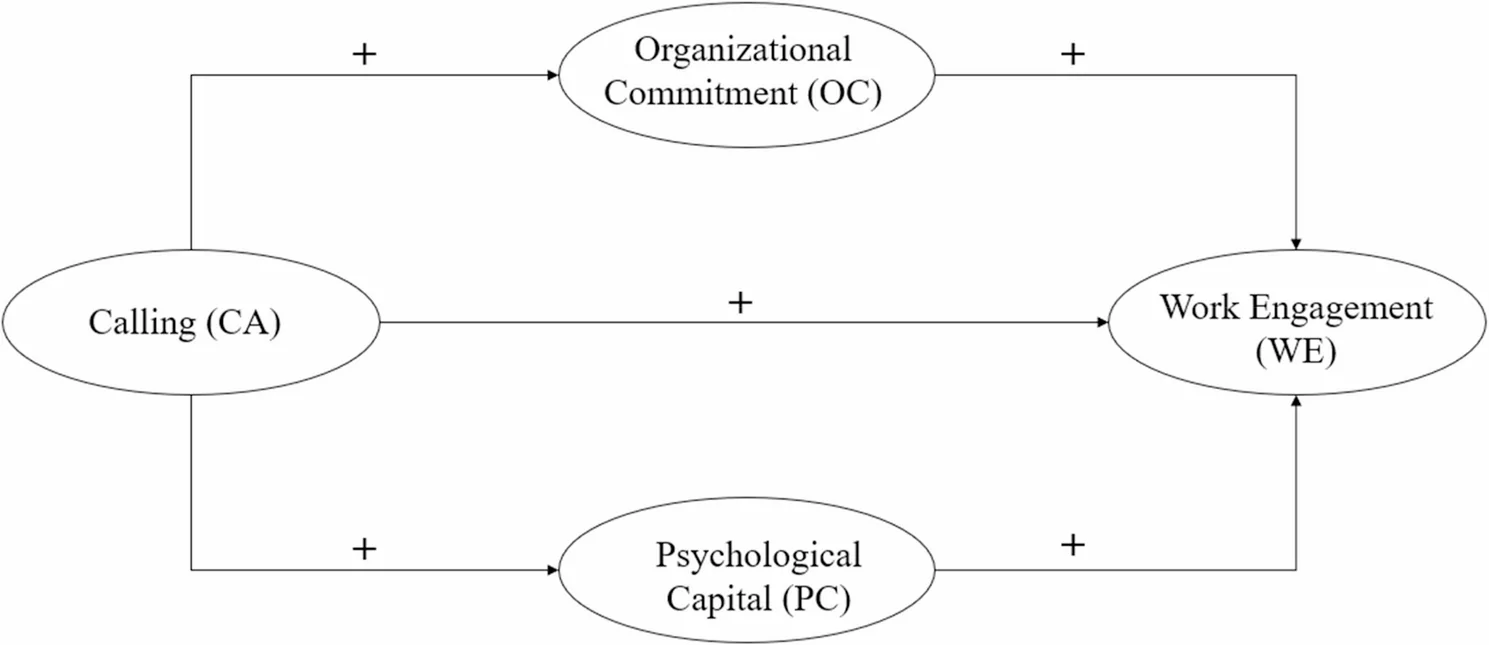
Theoretical model

Although numerous studies have examined the link between OC and work engagement in other fields, research in the nursing context remains limited. Cao found that the relationship between work engagement and CA was partially mediated by OC [34]. OC is typically categorized as comprising three components: continuance, affective, and normative commitment [40]. Continuance commitment refers to the perceived costs associated with leaving the organization [41]. Affective commitment is characterized by emotional attachment to the organization and identification with its goals and values [42]. Normative commitment reflects employees’ sense of obligation to remain with the organization based on internalized social norms [43]. According to the JD-R framework, this positive organizational attachment, as a valuable resource, can stimulate employees’ motivation and ultimately foster work engagement. Therefore, the following hypothesis is put forward (Fig. 1):

- H3: Organizational commitment mediates the relationship between calling and work engagement.

Indeed, empirical studies applying structural equation modeling to examine nurses’ work engagement remain limited. Therefore, it is crucial to examine the mediating roles of OC and PC in the correlation between CA and work engagement. The findings of this study can assist nursing managers in developing strategies to enhance nurses’ sense of CA, improving OC and PC, thus increasing work engagement and reducing burnout.

## Methods

### Study design

This research utilized a cross-sectional survey design involving nurses from a tertiary hospital in Guangzhou, China.

### Participants and sample size

Clinical nurses were recruited using convenience sampling. Participants were eligible if they met the following criteria: (A) certified registered nurses, (B) at least 1 year of clinical experience, and (C) provision of informed consent. Exclusion criteria were: (A) individuals not meeting the inclusion criteria, and (B) nurses not currently practicing. Participants were informed of the anonymity and confidentiality of the survey, as well as their right to withdraw at any time. For Structural Equation Modeling (SEM), a minimum sample size of 200 is generally recommended, or at least 5–10 times the number of estimated parameters [44]. With 13 observed variables, a total of 609 clinical nurses were included, meeting the recommended sample size requirements.

### Data collection

A total of 630 electronic questionnaires were collected between November and December 2021. The data were collected in late 2021, during the COVID-19 pandemic, a period when frontline nurses experienced substantially increased workload and psychological stress. Although a time interval exists between data collection and the preparation of this manuscript, the core theoretical relationships examined in this study reflect relatively stable psychological and organizational constructs. These relationships are therefore considered to remain relevant for current nursing practice and management.

Prior to the online survey, a designated investigator contacted nursing department directors to explain the research purpose. After obtaining verbal permission for data collection, the questionnaire link and informed consent information were distributed to each department. Nurses completed an online questionnaire assessing socio-demographic factors, CA, OC, PC, and work engagement. The questionnaires were distributed using the online platform Questionnaire Star (https://www.wjx.cn/). Submitted questionnaires were reviewed by the investigator, and data collection was completed within one month. Of the 630 questionnaires collected, 21 were excluded due to missing or invalid data, resulting in 609 valid responses (96.6%) included in the final analysis, demonstrating a high response rate.

Participants had a mean age of 33.79 ± 8.09 years (range 21–58), with the majority being female (95.7%) and married (62.2%). The proportion of female nurses in the sample (95.7%) is highly consistent with the national statistics for the nursing workforce in China. According to the National Health Commission, female nurses have consistently accounted for approximately 97% of all registered nurses in recent years [10]. This alignment supports the representativeness of the sample in terms of gender distribution. A total of 85.9% held a bachelor’s degree, and 32.3% had more than 15 years of work experience. Most participants reported a monthly income exceeding 1,385 USD. Regarding professional title, 61.7% were at the primary level or below. In terms of position and employment status, 76.8% were general duty nurses, and 54.0% were permanent staff. The demographic characteristics of the participants are presented in Table 1.

**Table 1:** Participants’ characteristics (*N* = 609)

	*N*(%)		*N*(%)
Age		Monthly income(USD)	
≦ 30	232(38.1)	415–692	88(14.4)
31–40	243(39.9)	693–1108	148(24.3)
≧ 41	134(22.0)	1109–1385	112(18.4)
Sex		> 1385	261(42.9)
Men	26(4.3)	Technical title	
Woman	583(95.7)	Primary or below	376(61.7)
Marital Status		Intermediate	224(36.8)
Unmarried	216(35.5)	Deputy senior or above	9(1.5)
Married	379(62.2)	Current position	
Divorced/Widowed	13(2.1)	General duty nurse	468(76.8)
Educational background		Specialist nurse	59(9.7)
Junior college degree	75(12.3)	Supervising nurse	57(9.4)
Undergraduate degree	523(85.9)	Head nurse	25(4.1)
Master’s degree or above	11(1.8)	Employment type	
Professional experience		Permanent staff	329(54.0)
1–3	123(20.2)	Contract staff	200(32.8)
4–9	144(23.6)	Temporary staff	80(13.1)
10–15	145(23.8)		
≧ 15	197(32.3)		

### Measurements

#### Measurements of calling

CA is described as a sustained and meaningful drive that motivates individuals to engage wholeheartedly in their work without anticipating additional external rewards [5].

A 12-item, unidimensional CA scale created by Dobrow was utilized in this research and has demonstrated high reliability in Chinese samples (Cronbach’s α = 0.947) [45]. Participants rated each item on a 5-point Likert scale ranging from 1 (strongly disagree) to 5 (strongly agree). The total score was calculated as the mean of all item responses, with higher scores indicating a stronger sense of CA.

#### Measurements of organizational commitment

OC is described as an individual’s inclination, obligation, or necessity to remain affiliated with an organization [11]. In this study, the Chinese version of the OC developed by Allen was utilized [46]. It is important to note that while the original theoretical framework posits three components (i.e., affective, continuance, and normative commitment) [40, 46], the Chinese version adapted for this study comprises five dimensions: affective, normative, ideal, opportunity, and economic commitment, as validated in previous Chinese nursing samples [47, 48]. Hence, the survey comprised 25 questions across five dimensions: affective, normative, ideal, opportunity, and economic commitment. Participants rated each item on a 5-point Likert scale ranging from 1 (strongly disagree) to 5 (strongly agree). The total score was calculated by summing all item responses, yielding a possible range of 25 to 125, with higher scores indicating greater OC. Previous studies have reported a Cronbach’s α of 0.67 for this scale in Chinese samples, indicating marginal reliability and validity [47, 48].

#### Measurements of psychological capital

PC is a key construct in positive psychology, referring to a positive psychological state that individuals develop over time [16]. The initial 24-item PC questionnaire was developed by Luthans [49]. In this study, the revised 20-item psychological capital questionnaire (PCQ-R) developed by Luo Hong [50] was used to assess nurses’ PC, demonstrating high internal consistency (Cronbach’s α = 0.923). The scale consists of four elements: self-efficacy, hope, optimism, and resilience. Items are rated on a 6-point Likert scale ranging from 1 (strongly disagree) to 6 (strongly agree). Higher scores indicate greater levels of PC.

#### Measurements of work engagement

Work engagement is defined as a positive, fulfilling work-related state characterized by vigor, dedication, and absorption [3]. The Utrecht Work Engagement Scale (UWES-9) was utilized to assess work engagement and demonstrated high internal consistency (Cronbach’s α = 0.95) [51]. Work engagement was assessed across three dimensions: vigor (3 items), dedication (3 items), and absorption (3 items). Items are rated on a 7-point Likert scale ranging from 0 (never) to 6 (every day). Mean scores are calculated for each dimension, with higher scores indicating greater work engagement.

### Data analysis

Data were analyzed using SPSS and Mplus. Initially, descriptive statistics and Pearson correlation analyses were conducted using SPSS 19.0 to examine the relationships among CA, OC, PC, and work engagement, and to assess the suitability for subsequent mediation analysis. Subsequently, SEM with maximum likelihood estimation was developed using Mplus 8.3 to examine the relationships among CA, OC, PC, and work engagement. Path analysis was conducted, and the bootstrap method with 5,000 resamples was utilized to estimate 95% confidence intervals for the mediation effects.

Model fit was evaluated using the following indices: Standardized Root Mean Square Residual (SRMR), Comparative Fit Index (CFI), Tucker-Lewis Index (TLI), and Root Mean Square Error of Approximation (RMSEA). Acceptable model fit was indicated by SRMR < 0.08, CFI and TLI ≥ 0.90, and RMSEA < 0.08.

Given the cross-sectional design and reliance on self-reported data, common method bias may be present. Hence, Harman’s single-factor test was conducted to assess the extent of common method bias. All measurement items from the CA, OC, PC, and work engagement scales were entered into an exploratory factor analysis. The results showed that the variance explained by the first factor was below the critical threshold of 40%. Therefore, common method bias was considered to be within an acceptable range.

## Results

### Correlations among calling, organizational commitment, psychological capital, and work engagement

Pearson correlation coefficients among the study variables are shown in Table 2. The results indicated that OC was positively related to PC (*r* = .41, *p*<.001), CA (*r* = .50, *p*<.001), and work engagement (*r* = .50, *p*<.001). PC demonstrated a positive correlation with CA (*r* = .47, *p*<.001) and work engagement (*r* = .47, *p*<.001). CA was positively correlated with work engagement (*r* = .52, *p*<.001).

**Table 2:** Correlations of key Studied Variables(*N* = 609)

Variables	OC	PC	CA	WE
OC	1			
PC	0.413**	1		
CA	0.500**	0.468**	1	
WE	0.500**	0.471**	0.515**	1

**P < .01. OC refers to organizational commitment, PC refers to psychological capital, CA refers to calling, and WE refers to work engagement (The same as below)

### Confirmatory factor analyses

Furthermore, the CFA results supported the discriminant validity of the four primary constructs (CA, OC, PC, and work engagement).

As shown in Table 3, the hypothesized four-factor model demonstrated the best fit to the data (χ2/df = 2.53, *p*<.001; CFI = 0.964, TLI = 0.959, RMSEA = 0.050, SRMR = 0.029), outperforming the three-factor model (χ2/df = 8.01, *p*<.001; CFI = 0.832, TLI = 0.814, RMSEA = 0.107, SRMR = 0.174), the two-factor model (χ2/df = 11.21, *p*<.001; CFI = 0.754, TLI = 0.730, RMSEA = 0.129, SRMR = 0.106), and the one-factor model (χ2/df = 14.73, *p*<.001; CFI = 0.668, TLI = 0.636, RMSEA = 0.150, SRMR = 0.107). These results provide strong support for the discriminant validity of the four constructs in the measurement model.

**Table 3:** Comparison of Measurement Models (*N* = 609)

Model	Description	χ2	df	χ2/df	CFI	TLI	RMSEA	SRMR
The baseline four-factor model	CA, OC, PC, WE	622.746	246	2.53	0.964	0.959	0.050	0.029
The three-factor model	CA + WE, OC, PC	1995.824	249	8.01	0.832	0.814	0.107	0.174
The two-factor model	CA + WE, OC + PC	2813.835	251	11.21	0.754	0.730	0.129	0.106
The one-factor model	CA + WE+OC + PC	3713.913	252	14.73	0.668	0.636	0.150	0.107

### Structural model assessment

#### Path analysis

**Fig. 2:**
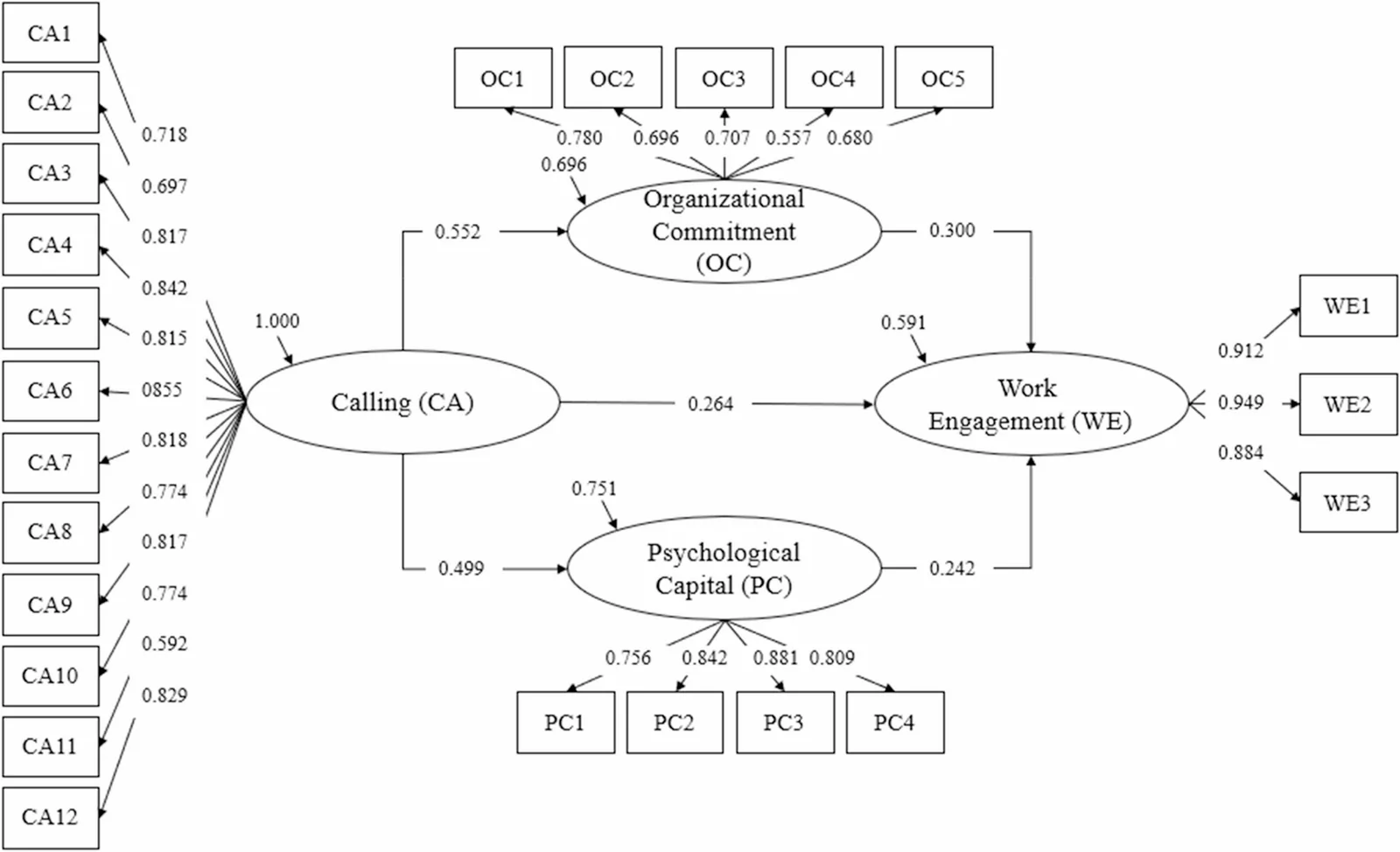
The mediating effect between calling and work engagement (*N* = 609)

SEM was conducted using Mplus 8.3 to examine the relationships among CA, OC, PC, and work engagement. As shown in Fig. 2, CA was positively associated with work engagement (β = 0.264, *p*<.001). Furthermore, CA was positively associated with OC (β = 0.552, *p*<.001) and PC (β = 0.499, *p*<.001). Additionally, both OC and PC correlate with work engagement (β = 0.300, *p*<.001; β = 0.242, *p*<.001). The model demonstrated a good fit to the data (χ2/df = 2.53, *p*<.001; CFI = 0.964, TLI = 0.959, RMSEA = 0.050, SRMR = 0.029). Moreover, as show in Table 4, the model explained 24.9%, 30.4%, and 40.9% of the variance in psychological capital, organizational commitment, and work engagement, respectively.

**Table 4:** Explained Variance (R²) of the Endogenous Constructs

Endogenous construct	*R*²	Explained variance
Psychological capital (PC)	0.249	24.9%
Organizational commitment (OC)	0.304	30.4%
Work engagement (WE)	0.409	40.9%

R² values were obtained as 1 minus the STDYX residual variances reported in the existing structural model figure

### Mediation analysis

Bootstrapping with 5,000 resamples was conducted in Mplus 8.3 to explore the mediating effects of OC and PC on the relationship between CA and work engagement. The bootstrapped estimates indicated that both direct and indirect pathways were statistically significant, as the 95% confidence intervals did not include zero. As shown in Table 5, the bootstrap results showed a total effect of 0.550 (SE = 0.031, 95% CI [0.483, 0.606]), a total indirect effect of 0.286 (SE = 0.031, 95% CI [0.228, 0.350]), and a direct effect of 0.264 (SE = 0.044, 95% CI [0.176, 0.348]). The specific indirect effects through organizational commitment (0.165, SE = 0.027, 95% CI [0.113, 0.219]) and psychological capital (0.121, SE = 0.021, 95% CI [0.084, 0.158]) were both supported because neither confidence interval included zero. These findings are consistent with a parallel mediation model.

**Table 5:** Bootstrapping Results for the Mediating

Effect	Path	Standardized effect (STDYX)	SE	Bootstrapping 95% CI
Total effect		0.550	0.031	0.483,0.606
Total Indirect effect		0.286	0.031	0.228,0.350
Direct	CA→WE	0.264	0.044	0.176,0.348
Indirect effect	CA→OC→WE	0.165	0.027	0.113,0.219
	CA→PC→WE	0.121	0.021	0.084,0.158

## Discussion

The current study empirically demonstrates the roles of CA, PC, and OC in contributing to work engagement among nurses in China. Previous studies have primarily focused on bivariate relationships, often overlooking their simultaneous effects. The main contribution of this study is to provide novel insights into the parallel mediating roles of PC and OC in the relationship between CA and work engagement within the job demands–resources (JD–R) framework. By utilizing structural equation modeling and simultaneously incorporating multiple variables, this study provides a more comprehensive and robust analytical framework.

The study indicates that a higher degree of CA is associated with greater work engagement, consistent with previous studies conducted in Western contexts [6]. According to the JD-R model, CA functions as a personal resource that increases work engagement by increasing perceived job meaningfulness and fostering intrinsic motivation. Nurses who perceive their work as a CA are more likely to internalize professional values and experience harmonious passion [48], which in turn corresponds to sustained vigor and absorption in daily tasks. This form of harmonious passion, which enables individuals to engage freely in their work, has been consistently associated with positive outcomes such as job satisfaction, creativity, and collaborative relationships with colleagues [52]. Thus, individuals who perceive work as a CA are more likely to integrate work into their identity, fostering stronger engagement in their professional roles. Therefore, nurses with a strong sense of CA are more likely to exhibit higher levels of work engagement. Building on this direct relationship, the present study further examines whether this association is mediated by PC and OC.

Drawing on the JD-R framework, this study provides novel evidence regarding the mediating role of PC in the relationship between CA and work engagement. The findings extend existing literature by demonstrating that personal psychological resources play a critical role in linking calling to work engagement. The significant indirect effects further indicate that CA influences work engagement through employees’ PC. A stronger sense of CA can enhance individuals’ PC, thereby contributing to higher levels of work engagement. Consistent with prior research, PC has been shown to significantly predict work engagement [53]. Hence, as a mediating mechanism, CA translates the positive effects of calling into higher work engagement among nurses. PC comprises four core components: self-efficacy, hope, optimism, and resilience. Nurses with a strong sense of CA are more likely to pursue tough goals.

CA is a vital personal asset that contributes to nurses’ self-efficacy. Self-efficacy, a vital component of PC, correlates with individuals’ cognitions, behaviors, and emotional regulation. Past studies have demonstrated a positive correlation between self-efficacy and work engagement [54, 55]. These findings suggest that CA are associated with work engagement through self-efficacy, a key dimension of PC. Nurses with high self-efficacy are better able to regulate their emotions, maintain a positive affective state, and effectively utilize PC to cope with challenges and mobilize job resources. This, in turn, leads to higher levels of effort, motivation, and work engagement, particularly in terms of vigor, absorption, and dedication. From a managerial perspective, both CA and PC should be considered in efforts to enhance nurses’ work engagement. Supporting nurses with a strong sense of CA may enhance their self-efficacy and PC, align their personal values with their CA, and ultimately increase work engagement while reducing negative outcomes such as turnover intention. In addition to PC, OC also serves as a key mediator linking CA and work engagement, operating at the organizational rather than the individual level.

The relationship between CA and work engagement is mediated by OC, as CA is positively associated with OC, which in turn affects work engagement. Previous studies have indicated that nurses with a strong sense of CA exhibit higher levels of work-related well-being, enthusiasm, life satisfaction, and commitment to both their profession and organization. This mediating effect can be further understood by considering the affective component of the process. Nurses with a strong sense of CA are more likely to develop affective commitment, as they perceive alignment between their personal values and organizational goals. In addition, affective commitment fosters discretionary effort and higher levels of work engagement. These results align with research conducted in the past [18]. Consistent with this, Xie [56] found that a strong sense of vocation helps individuals cope effectively with stress and anxiety. Prior research has identified meaningful work, organizational support, and occupational identification as key mediators linking CA to favorable outcomes such as OC, job satisfaction, and intention to remain [57, 58]. A longitudinal study by Hakanen [59] further highlighted a strong correlation between OC and work engagement. OC plays a crucial role in shaping the relationship between employees and their organization, and assessing commitment can inform managerial decisions that influence organizational effectiveness. Enhancing nurses’ organizational commitment may contribute to increased work engagement. Nursing managers should adopt effective leadership strategies to foster employees’ sense of belonging. Organizations should provide adequate support to help nurses achieve their professional goals and enhance their competencies, thereby promoting higher levels of work engagement.

These findings provide practical implications for multiple stakeholders. For nurse managers, the results suggest that fostering a sense of calling among clinical nurses may serve as a valuable strategy to enhance work engagement. This can be accomplished by organizing frequent team meetings where nurses get an opportunity to discuss the meaningful patient care experiences, thus emphasizing the importance of their everyday activities. Moreover, nurse managers may also consider implementing resilience training programs along with goal setting activities to enhance the psychological capital of nurses. For hospital administrators and policymakers, the findings underscore the importance of creating an organizational environment that supports affective commitment. The strategies can be seen through involving the nurses in making clinical decisions, valuing their professional achievements, and developing career progression paths. Organizational-level efforts like these would definitely improve the emotional bond that nurses share with the hospital and consequently increase their engagement at work. For nurse educators and academic institutions, the results highlight the value of incorporating calling-oriented content into nursing curricula. Educators could incorporate activities aimed at nurturing reflection in the learning process through which nursing students could discover their professional identity and cultivate a strong sense of purpose in their patient care work. Cultivating a sense of calling early in one’s career could be instrumental in sustaining engagement.

## Conclusion

The present study indicates that CA is an important correlate of work engagement among Chinese nurses, exerting both direct effects and indirect effects via PC and OC. This study contributes to the JD-R literature by demonstrating that personal resources (e.g., CA and PC), and job-related resources (e.g., OC) jointly promote work engagement. These findings highlight the need for targeted interventions to enhance CA, PC, and OC. Accordingly, nurse managers are encouraged to implement targeted strategies to enhance nurses’ sense of CA (e.g., sharing meaningful patient care experiences), develop PC through resilience training and goal-setting interventions, and strengthen affective commitment by aligning organizational values with professional nursing ideals.

The research has several limitations. Firstly, the study employed a cross-sectional design, with all data collected at a single time point. Although the structural equation model supported the hypothesized relationships among CA, PC, OC, and work engagement, causal inferences cannot be established, and reverse causality or unobserved confounding variables cannot be ruled out. Therefore, future research should adopt longitudinal or experimental designs to better examine temporal relationships and causal mechanisms among these variables. Secondly, the sample was drawn from a single center, which might affect the generalizability of results obtained through this study. The participants may not be representative of nurses working in community hospitals, primary care settings, or other geographic regions across China. Hence, future multi-center studies with probability sampling are needed to confirm and extend these findings. Thirdly, the Cronbach’s α for the organizational commitment scale was 0.67 in this sample, which is lower than the typical cut-off point of 0.70. This marginal reliability may have attenuated the observed associations. In the future research on this topic, other measurement methods of organizational commitment are suggested. Moreover, while this study focused on the correlation between CA and work engagement, as well as the mediating roles of PC and OC, the potential interaction or reciprocal relationship between PC and OC was not examined. Hence, future research should further investigate the relationship between PC and OC within the JD-R framework. Furthermore, to align with the JD-R framework and maintain model parsimony, this study employed composite scores of PC and OC in the analysis. However, this approach may obscure potential differential effects across specific dimensions. Future research should examine the distinct effects of specific dimensions, thereby providing more nuanced theoretical insights and practical implications.

## Data Availability

All data was in the manuscript.

## Abbreviations

SEM: Structural Equation Modeling
JD-R: Job Demands-Resources
CFI: Comparative Fit Index
TLI: Tucker-Lewis Index
RMSEA: Root Mean Square Error of Approximation
SRMR: Standardized Root Mean Square Residual
OC: Organizational Commitment
PC: Psychological Capital
CA: Calling
WE: Work Engagement
